# LncRNA HCP5 Facilitates the Progression of Ovarian Cancer by Interacting with the PTBP1 Protein

**DOI:** 10.1007/s10528-023-10558-8

**Published:** 2023-12-10

**Authors:** Jian Shou, Chuanling Zhang, Xiaoyu Zheng, Yaowei Li, Peng Wu, Long Chen, XiuJun Wei

**Affiliations:** 1 Department of Gynecology, Affiliated Xiaoshan Hospital, Hangzhou Normal University, No. 728, North Yucai Road, Beigan Street, Xiaoshan District, Hangzhou, 311200 Zhejiang China; 2 Department of Translational Medicine Laboratory, Affiliated Xiaoshan Hospital, Hangzhou Normal University, No. 728, North Yucai Road, Beigan Street, Xiaoshan District, Hangzhou, 311200 Zhejiang China

**Keywords:** Ovarian cancer, HCP5, PTBP1, LncRNA

## Abstract

Ovarian cancer (OC) is a major gynecological malignancy with an annually increasing morbidity that poses a significant threat to the health of women worldwide. Most OC patients are diagnosed at an advanced stage. It is an urgent task to search for biomarkers for the diagnosis and treatment of OC. The lncRNA HCP5 (HCP5) was recently identified as an oncogene in several malignant tumors. However, the function of HCP5 in OC has rarely been reported. Herein, the levels of HCP5 and PTBP1 were found to be markedly increased in malignant OC tumor tissues and OC cell lines. In HCP5-silenced SKOV-3 and HEY cells, cell viability was markedly decreased, and the apoptosis rate was significantly increased, with more cells exhibiting G0/G1 arrest and increased expression of cleaved caspase-3 and cleaved caspase-9. Furthermore, the number of migrated cells, number of invaded cells, and migration distance were notably decreased by the knockdown of HCP5 in SKOV-3 cells and HEY cells. In the xenograft model established with SKOV-3 cells, the number of lung metastases, tumor growth, and Ki67 expression in tumor tissues were markedly decreased by the knockdown of HCP5, accompanied by an increased percentage of TUNEL-positive cells. HCP5 was found to be localized in the nucleus, and the interaction between HCP5 and PTBP1 was verified by RNA pull-down and RNA immunoprecipitation assays. Furthermore, in HCP5-overexpressing OC cells, the impacts of HCP5 on cell proliferation and apoptosis were significantly attenuated by the knockdown of PTBP1. Collectively, these results indicate that HCP5 facilitates the progression of OC by interacting with the PTBP1 protein.

**Supplementary Information:**

The online version contains supplementary material available at 10.1007/s10528-023-10558-8.

## Introduction

OC is one of three major gynecological malignancies with an annually increasing morbidity, and it has the highest mortality among malignant tumors of the female reproductive system. In the United States, approximately 22,280 cases of OC are diagnosed and OC causes approximately 14,240 deaths annually. (Siegel et al. 2022). In China, 52,100 cases of OC and 22,500 deaths from OC in one year have been reported (Chen et al. 2016). Ovarian cancer is prone to relapse, and recurrence or chemotherapeutic resistance is observed in approximately 75% of OC patients (Ozols et al. 2003). Therefore, novel therapeutic targets and prognostic indicators are urgently needed, and the study of the function and expression regulation of related genes that play important roles in OC is, thus, a critical task.

LncRNAs are noncoding RNAs with a length of more than 200 nucleotides and are involved in the regulation of epigenetics, the cell cycle, cell differentiation, and other processes (Bridges et al. 2021). LncRNAs have been found to be involved in the development of malignant tumors, including OC (Li et al. 2016). Hongbo Wang et al. used high-throughput RNA sequencing to identify 19 differentially expressed lncRNAs, 99 differentially expressed circRNAs, 28 differentially expressed miRNAs, and 304 differentially expressed mRNAs between HPV16-mediated ovarian squamous cell carcinoma and adjacent nontumor tissues, and this study laid the foundation for functional research on coding and noncoding RNAs in OC (Wang et al. 2017). Burk RD et al. reported a large-scale study on the genetic characteristics of OC based on whole exome sequencing, revealing the mutated genes related to OC and the relationship between the lncRNA BCAR4 and sensitivity to the targeted drug lapatinib (Cancer Genome Atlas Research N et al. 2017). Furthermore, it is asserted that in HPV16-related OC, the HPV E7 protein positively regulates the expression of the lncRNA HOTAIR, while reducing HOTAIR expression upregulates HOXD10 to affect the proliferation of tumor cells (Sharma et al. 2015). Screening for novel lncRNAs with functions in OC is critical for the diagnosis and prognostic prediction of OC.

The gene-encoding HCP5, or *Homo sapiens* HLA complex P5, is located at chr6:31,400,702–31,465,809. Currently, HCP5 is reported to be involved in the progression of multiple malignant tumors. Tse KP et al. (Tse et al. 2011) analyzed the cervical nodal volume (CNV) in patients with nasopharyngeal carcinoma and found that 8 CNV regions in patients with nasopharyngeal carcinoma were significantly changed compared with those in the normal population and that single copy deletion of HCP5 occurred in the stained 6p21.3 region and was significantly related to the occurrence of nasopharyngeal carcinoma. In a study of HCV-carrying liver cancer patients in Switzerland (Lange et al. 2013), HCP5 was found to be a susceptibility locus for HCV-associated hepatocellular carcinoma. In the study of glioma, HCP5 was found to be correlated with the migration and proliferation of glioma cells. The expression of the RUNT-related transcription factor RUNX1 is inhibited by the knockdown of HCP5, and RUNX1 binds to microRNA-139 to affect the metastasis and proliferation of tumor cells. Li reported that the HCP5 level was significantly related to overall survival in OC patients (Li and Zhan 2019). However, the specific role of HCP5 in OC remains unknown. The present study explored the function and potential regulatory mechanism of HCP5 in OC to identify valuable biomarkers for the diagnosis and prognosis of OC.

## Materials and Methods

### Tumor Tissues

Ten pairs of malignant tumor tissues and paracarcinoma tissues were collected from 10 OC patients, while 10 pairs of benign tumor tissues were collected from non-OC patients (including patients with endometriotic cysts, mucinous cystadenoma, and ovarian teratoma). Patients were recruited from Oct 25th, 2021, to Feb 07th, 2023, in Xiaoshan Hospital of Zhejiang Province. All patients agreed to the use of their samples in scientific research, and informed consent was given orally.

### Cell lines and Treatments

A2780 cells (iCell-h004) and HEY cells (iCell-h364) were purchased from iCell (China). SKOV-3 cells (HTB-77) were purchased from ATCC (USA). HOSE11-12 cells were obtained from Shanghai Zeye Biotechnology Co., Ltd. (AC340096, China) and were incubated in DMEM supplemented with 10% FBS in 5% CO2 and 37 °C.

### RT‒PCR

Tumor tissues or cells were collected for extraction of total RNA using an RNA extraction kit (B618583-010, Sangon Biotech, China). Subsequently, cDNA synthesis was performed utilizing the RT‒PCR reverse transcription kit (CW2569, CWBIO, China) prior to PCR amplification in a thermal cycler (LightCycler® 96, Roche, Switzerland) using the SYBR Green qPCR kit (11201ES08, Yeasen, China). The internal reference gene was β-actin, and relative gene expression levels were determined utilizing the 2^−ΔΔCt^ method. The sequences are shown in Table 1.

**Table 1:** Sequences of primers

Gene	Forward primer	Reverse primer
HCP5	GACTCTCCTACTGGTGCTTGGT	CACTGCCTGGTGAGCCTGTT
PTB	AGCGCGTGAAGATCCTGTTC	CAGGGGTGAGTTGCCGTAG
β-actin	CATGTACGTTGCTATCCAGGC	CTCCTTAATGTCACGCACGAT

### Immunohistochemical Assay

Tumor tissues were removed, fixed with 4% paraformaldehyde, dehydrated through an ethanol gradient, embedded in paraffin, and sectioned (5 μm). The paraffin sections were dewaxed, washed with water, and incubated with 1% methanol/hydrogen peroxide for 10 min to block peroxidase activity. After washing using PBS buffer, the tissues were incubated with citrate buffer (pH 6.0) for 10 min prior to incubation in a microwave oven for 10 min. After the tissues were cooled to room temperature, they were blocked with goat serum for 20 min, and primary antibodies against HCP5 (1:100, DF15552, Affinity, USA) or PTBP1 (1:100, DF6644, Affinity, USA) were then added for incubation at 4 °C overnight. Then, a HRP-conjugated secondary antibody (1:5000, ab97080, Abcam, UK) was added for incubation at 37 °C for 20 min prior to the addition of DAB solution. After dehydration and sealing, images were acquired using a microscope (E100, Nikon, Japan).

### Knockdown of HCP5 or PTBP1 in SKOV-3 and HEY Cells

To knock down the expression of HCP5 or PTBP1 in OC cells, SKOV-3 and HEY cells were transfected with three siRNAs targeting HCP5 (si-HCP5-1, si-HCP5-2, and si-HCP5-3) or si-PTBP1 with Lipofectamine 3000 (Invitrogen, USA) for 48 h. The transfection efficiency was determined using RT‒PCR, and the siRNA with the highest transfection efficiency was chosen for subsequent experiments. The sequences of the siRNAs are shown in Table 2.

**Table 2:** Sequences of siRNAs

Sequence name		Sequence (5′–3′)
si- LncHCP5-1	sense	5′ GGAUCAGGAUCUAUUACCUTT 3′
	Anti-sense	5′ AGGUAAUAGAUCCUGAUCCTT 3′
si- LncHCP5-2	sense	5′ CAGGUAAUCUAAGGAGAGUTT 3′
	Anti-sense	5′ ACUCUCCUUAGAUUACCUGTT 3′
si- LncHCP5-3	sense	5′ GUUCUUCCUACUGAGAUUATT 3′
	Anti-sense	5′ UAAUCUCAGUAGGAAGAACTT 3′
si- PTB	sense	5′ CCAAGAACTTCCAGAACATAT 3′
	Anti-sense	5′ ATATGTTCTGGAAGTTCTTGG 3′

### Establishment of HCP5-Overexpressing SKOV-3 and HEY Cells

The HCP5- overexpression vector (oe-lnc RNA HCP5) was designed, obtained, and packaged in adenoviral particles. Adenoviral particles containing oe-lnc RNA HCP5 were used to infect SKOV-3 and HEY cells for 48 h, with empty vector (oe-NC) as the negative control.

### CCK-8 Assay

After seeding cells in a 24-well plate for 24 h, 10 μL CCK-8 reagent was added, and the cells were cultured for 4 h prior to measurement of the optical density at 450 nm with a microplate reader (CMaxPlus, MD, USA).

### Flow Cytometric Analysis of Apoptosis

Cells were seeded in 6-well plates and incubated at 37 °C for 48 h prior to centrifugation at 300 × g for 5 min. Cells were collected and resuspended in serum-free medium and approximately 10 μL of Annexin V reagent (556547, BD, USA) and 5 μL of PI reagent (556547, BD, USA) prior to incubation for 10 min at room temperature in the dark. Then, the cell suspension was mixed with PBS buffer in a flow tube and analyzed by flow cytometry (NovoCyte, Agilent, USA) for analysis of apoptosis.

### Flow Cytometric Analysis of the Cell Cycle

The cell suspension was centrifuged at 1500 rpm for 3 min, and the supernatant was discarded prior to the addition of 1 mL PBS. After centrifugation at 1500 rpm, the supernatant was discarded. Then, 1 mL DNA staining solution and 10 μL permeabilization solution (550825, BD, USA) were added prior to vortexing for 5–10 s and incubation at room temperature in the dark for 30 min. Finally, the cell suspension was mixed with PBS buffer in the flow tube, and the cell cycle was analyzed by flow cytometry (NovoCyte, Agilent, USA).

### Western Blotting

Cells were seeded in a 35 mm dish and incubated for 1 d. The cell lysate was added and cultured for 30 min at 4 °C. After centrifugation at 12 000 rpm for 10 min, the supernatant was collected, and the protein concentration was quantified by the BCA method. Total protein was separated by SDS‒PAGE and transferred to a PVDF membrane, and the membrane was blocked with 5% skim milk powder for 1.5 h. Then, an anti-cleaved caspase-3 (1:1000, AF7022, Affinity, USA), anti-cleaved caspase-9 (1:1000, AF5240, Affinity, USA), or anti-β-actin (1:10,000, 81,115–1-RR, Proteintech, USA) antibody was added for incubation at 4 °C overnight. After washing, the membrane was incubated with the secondary antibody (1:2000, 7076, CST, USA) and was then exposed to ECL solution. ImageJ software was used for grayscale analysis.

### Transwell Assay

Tumor cells were collected, counted, and seeded into a transwell insert (3422, Corning, USA) at a density of 1.5 × 10 (Li et al. 2016) cells/well. The lower chamber was filled with medium containing 20% FBS. Cells were then cultured in serum-free medium in the upper chamber for 24 h at 37 °C and 5% CO_2_, and cells remaining in the upper chamber were removed by wiping. Subsequently, cells in the lower chamber were stained with crystal violet and counted under an optical microscope (AE2000, Motic, China).

### Invasion Assay

Matrigel (356234, BD, USA) was diluted with serum-free DMEM at a ratio of 3:1, and 30 μL of the mixture was used to evenly coat the membrane in the Transwell insert prior to incubation at 4 °C overnight. After treatment, the Transwell inserts were placed into a 24-well culture plate, with cells added into the upper compartment prior to incubation for 24 h in a 5% CO_2_ incubator at 37 °C. The Matrigel substrate and the cells on the bottom surface of the membrane in the upper chamber were removed with cotton swabs, and the remaining cells were then fixed with 4% paraformaldehyde for 10 min. After staining with 0.1% crystal violet solution for 30 min, images were acquired using an optical microscope (AE2000, Motic, China), and the cells that invaded into the lower chamber was counted.

### Wound Healing Assay

After the cells reached confluence, a Pasteur pipette was used to scrape a linear wound, and the scratch width was measured. Following 24 h of incubation, the detached cells and debris were removed using PBS, and the scratch width was measured to calculate the percentage of wound closure.

### Animals and Xenograft Model

Twelve female nude mice (7–9 weeks) were purchased from Shanghai SLAC Laboratory Animal Co., Ltd. (China). After one week of adaptive feeding, the animals were randomly divided into 2 groups: si-NC and si-HCP5. A total of 5 × 10^6^ cells were inoculated subcutaneously into the rear axillary region in a volume of 0.25 mL/mouse. In the si-NC group, si-NC-transfected SKOV-3 cells were injected, while in the si-HCP5 group, si-HCP5-transfected SKOV-3 cells were injected. The tumor volume was recorded once a week for 4 weeks. Tumors were weighed and sampled at the end of the experiments.

### HE Staining

Lung tissues and tumor tissues were fixed in 4% paraformaldehyde solution for 1‒2 h, dehydrated in a gradient of 70% to 100% ethanol, cleared in xylene, embedded in paraffin, and sectioned. The paraffin sections were deparaffinized with xylene; immersed in xylene for 10 min, 100% ethanol for 5 min, 90% ethanol for 5 min, 80% ethanol for 5 min, 70% ethanol for 5 min, and distilled water for 5 min; stained with hematoxylin for 10 min; washed with distilled water for 10 min; differentiated in 1% hydrochloric acid for several seconds; and counterstained with eosin. The sections were dehydrated, dried, sealed with neutral gum, and observed under a microscope (E100, Nikon, Japan). Lung metastases were counted in the lung tissue of each animal.

### TUNEL Assay

The apoptosis rate of cells in tumor tissue was evaluated by the TUNEL assay using a commercial kit (C1090, Beyotime, China). Sections were blocked using 5% BSA for 1 h prior to incubation with TUNEL solution for 10 min. After washing, DAPI-staining solution was added for a 5 min incubation. TUNEL-positive cells appeared brown and yellow. Five fields were randomly observed under a light microscope (Nikon Eclipse Ci-L, Nikon, Japan). The number of positive cells was determined by an image digital analysis system.

### Immunofluorescence Assay

After baking in an oven at 62 °C for 1 h, the sections were dewaxed, hydrated, and washed using PBS prior to the addition of 1 mM Tris–EDTA (pH = 9.0) for antigen retrieval. After blocking using goat serum for 20 min at 37 °C, the sections were incubated with a primary antibody against Ki67 (1:300, AF0198, Affinity, USA) at 4 °C overnight prior to the addition of a fluorescently labeled secondary antibody (1:500, ab150079, Abcam, UK) for 60 min at 37 °C. DAPI solution was then added, and after dehydration and sealing, images were acquired using a fluorescence microscope (Nikon, Japan).

### Fluorescence In Situ Hybridization (FISH) Assay

Cells were fixed in 4% paraformaldehyde for 15 min at room temperature and then permeabilized in precooled PBS containing 0.5% Triton X-100 for 5 min. The cells were washed three times for 10 min each in PBS and rinsed once in 2 × SSC buffer. Hybridization was performed with probes for HCP5, U6, and 18S (GemmaPharma, China) for 12 to 16 h at 37 °C. Cells were then washed sequentially with 4 × SSC buffer, 2 × SSC buffer, and 1 × SSC buffer. Finally, the cells were counterstained with DAPI for 10 min and visualized by laser confocal microscopy (LSM880, Zeiss, Germany).

### RNA Pull-Down Assay

Two microliters of DNase I was added to an EP tube and incubated at 37 °C for 15 min to remove DNA from the reaction system. The reaction was terminated by adding 2 µl 0.2 M EDTA (pH = 8.0). One microgram of biotin-labeled RNA was taken, and an appropriate amount of Structure Buffer was added to allow the formation of RNA secondary structures. The RNA was then heated at 95 °C for 2 min, placed in an ice bath for 3 min, and cooled for 30 min. The magnetic beads were washed 3 times using RIP Wash Buffer, and resuspended in 50 µl RIP Wash Buffer and added to biotin-labeled and denatured HCP5 RNA, and incubated overnight at 4 °C The mixture was then centrifuged at 3000 rpm for 1 min to remove the supernatant. The cell lysate was added to the magnetic bead-RNA mixture, and an appropriate amount of RNase inhibitor was added to the lysate. The incubated magnetic bead-RNA‒protein mixture was centrifuged at low speed, and the supernatant was recovered and rinsed 3 times using RIP Wash Buffer. SDS loading buffer (5 ×) was added to the samples prior to denaturation at 95 °C for 10 min and loading onto SDS‒PAGE gels. The target bands were excised to measure the expression of PTBP1 using Western blotting.

### RNA Immunoprecipitation

After cell lysis, the detection antibody was added, and the working concentration of the antibody was 8 μg per reaction system. After incubation at 4 °C overnight, the antibody was rewarmed at room temperature for 1 h. Protein G magnetic beads were added to capture the complexes, and the buffer was washed to extract RNA. The level of HCP5 was measured by RT‒PCR.

### Statistical Analysis

Data are shown as the mean ± SD values and were analyzed using one-way ANOVA with Tukey’s test. *p* < 0.05 was considered to indicate a statistically significant difference.

## Results

### HCP5 was Upregulated in Malignant Tumor Tissues of OC Patients

**Fig. 1:**
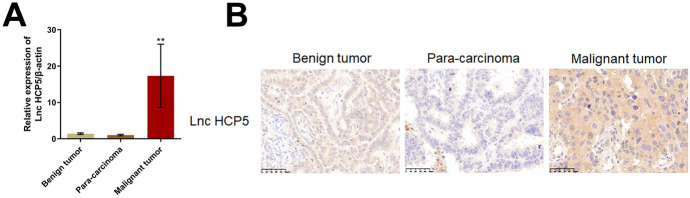
The level of HCP5 was markedly increased in malignant tumor tissues of OC patients. **A** The expression of HCP5 in benign tumor tissues, malignant tumor tissues, and paracarcinoma tissues was measured by RT‒PCR (**p < 0.01 vs. benign tumor tissues). **B** The expression of HCP5 in benign tumor tissues, malignant tumor tissues, and paracarcinoma tissues was evaluated by an immunohistochemical assay

To explore the potential function of HCP5 in OC, benign tumor tissues were collected from non-OC patients, while malignant tumor tissues and paracarcinoma tissues were collected from OC patients. As shown in Fig. 1A, compared to that in benign tumor tissues, the HCP5 level was markedly increased in malignant tumor tissues of OC patients and slightly changed in paracarcinoma tissues of OC patients. The results of the immunohistochemical assay also confirmed that HCP5 was upregulated in malignant tumor tissues compared to paracarcinoma tissues and benign tumor tissues (Fig. 1B).

### The HCP5 Level was Increased in OC Cell Lines

**Fig. 2:**
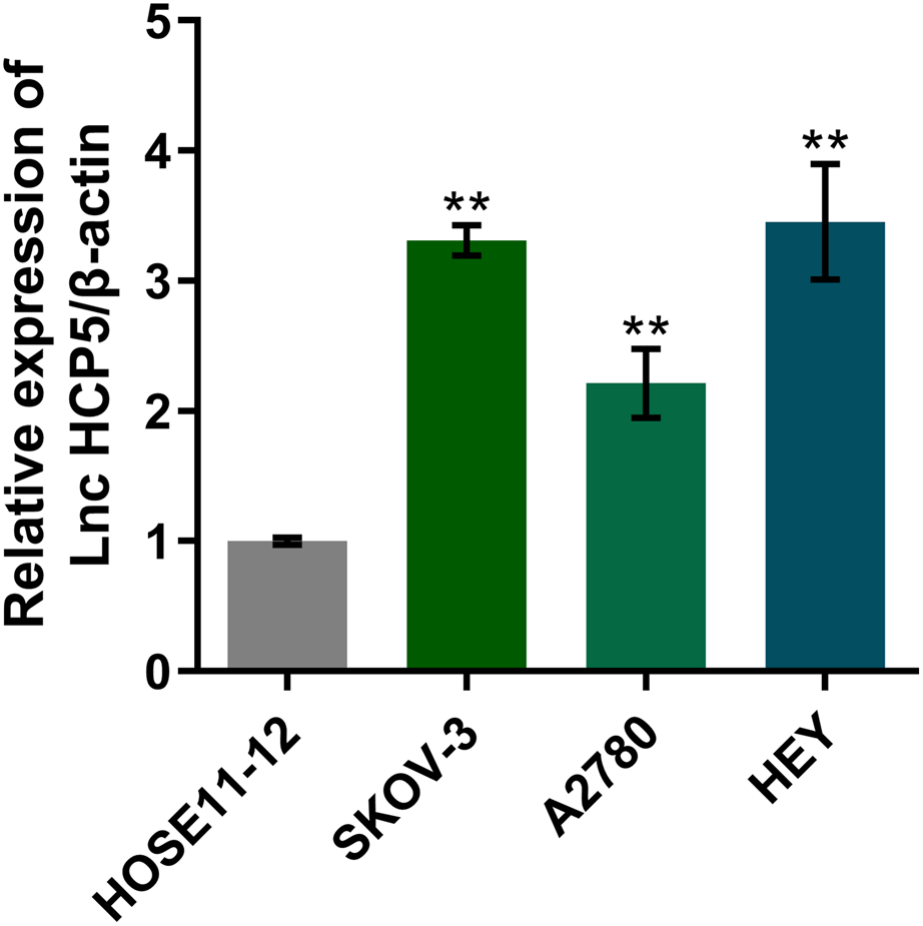
The HCP5 level was increased in OC cell lines. The expression of HCP5 in HOSE11-12, SKOV-3, A2780, and HEY cells was measured by RT‒PCR (**p < 0.01 vs. HOSE11-12)

To further confirm the expression of HCP5 in OC cell lines, 3 OC cell lines and a human ovarian epithelial cell line, HOSE11-12, were examined. As shown in Fig. 2, compared to that in HOSE11-12 cells, the HCP5 level was markedly elevated in SKOV-3, A2780, and HEY cells. Higher expression of HCP5 was observed in HEY cells and SKOV-3 cells. Therefore, SKOV-3 cells and HEY cells were chosen for subsequent assays.

### HCP5 was Successfully Knocked Down in SKOV-3 Cells and HEY Cells

**Fig. 3:**
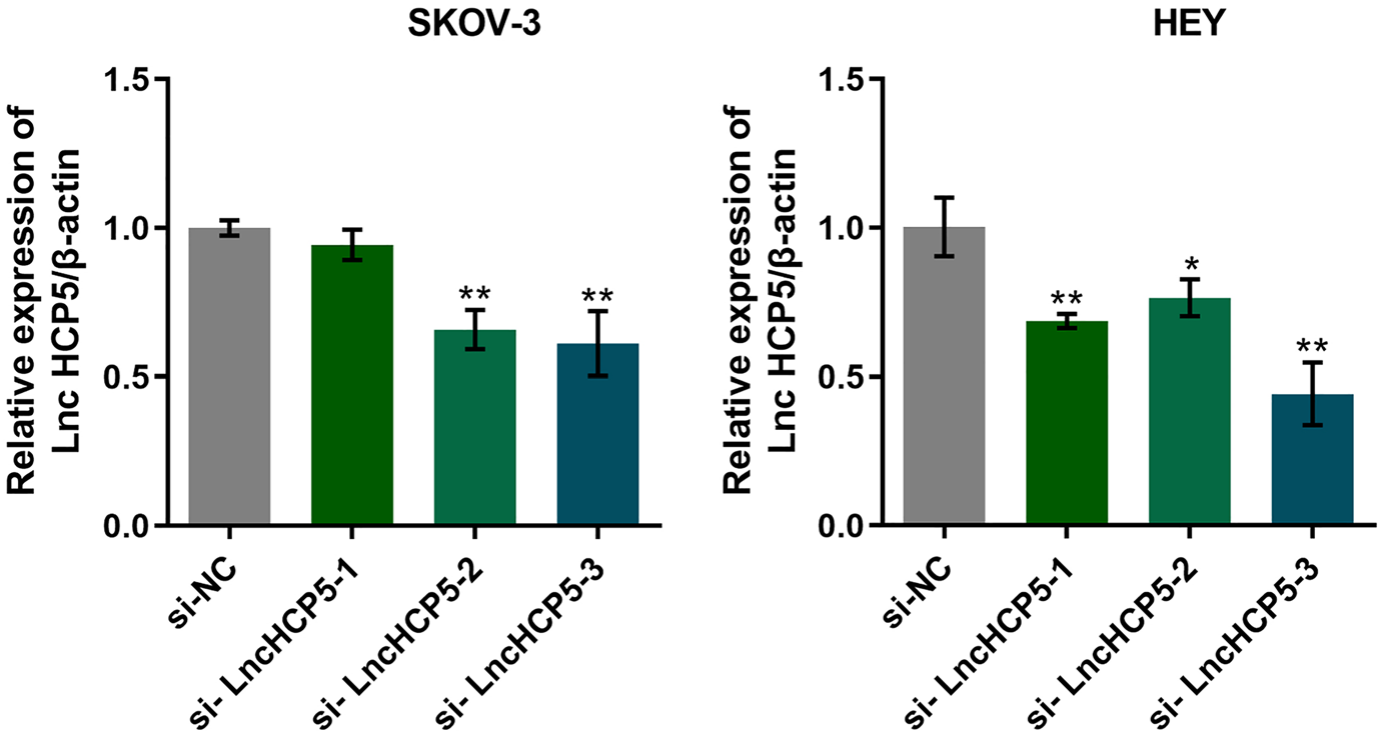
HCP5 was successfully silenced in SKOV-3 cells and HEY cells. The expression of HCP5 in siRNA-transfected SKOV-3 and HEY cells was measured by RT‒PCR (*p < 0.05 vs. si-NC, **p < 0.01 vs. si-NC)

To knock down HCP5 in OC cells, SKOV-3 cells and HEY cells were transfected with 3 siRNAs, with si-NC as the negative control. As shown in Fig. 3, compared to that in the si-NC-transfected counterparts, the HCP5 level was notably decreased in si-HCP5-1-, si-HCP5-2-, and si-HCP5-3-transfected SKOV-3 cells and HEY cells, with si-HCP5-3 showing the highest knockdown efficiency. Therefore, si-HCP5-3 was chosen for subsequent assays.

### Silencing of HCP5 Facilitated the Apoptosis and Suppressed the Proliferation of OC Cells

**Fig. 4:**
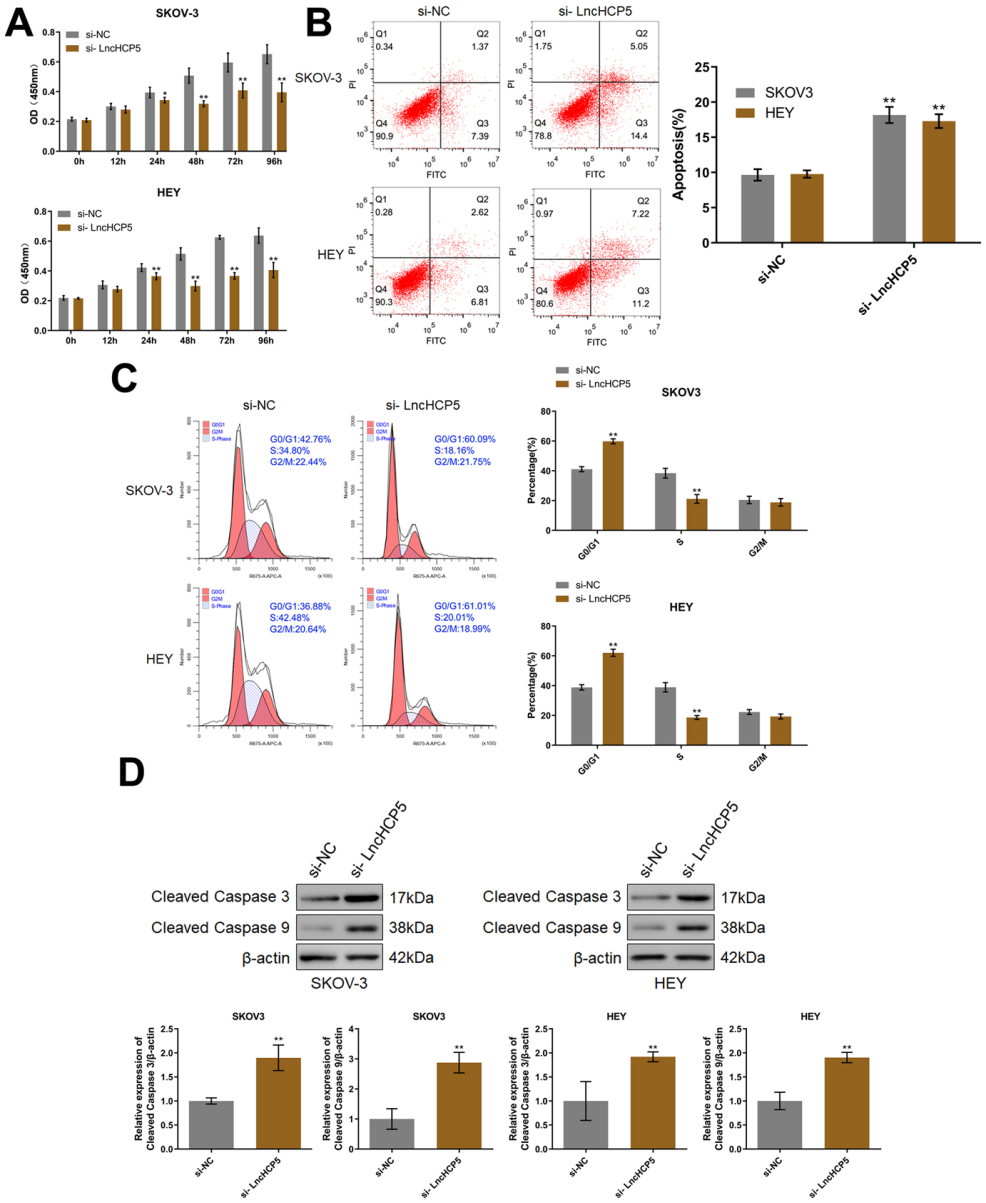
Silencing of HCP5 facilitated the apoptosis and suppressed the proliferation of OC cells. **A** The viability of SKOV-3 and HEY cells was checked by a CCK-8 assay. **B** Apoptosis in SKOV-3 and HEY cells was detected by flow cytometry. **C** The cell cycle in SKOV-3 and HEY cells was analyzed by flow cytometry. **D** The levels of cleaved caspase-3 and cleaved caspase-9 were measured by Western blotting (*p < 0.05 vs. si-NC, **p < 0.01 vs. si-NC)

The proliferation, cell cycle distribution, and apoptosis rate of si-HCP5-transfected OC cells were evaluated. In both SKOV-3 cells and HEY cells, when the incubation duration was longer than 24 h, cell viability was markedly decreased after the transfection of si-HCP5 (Fig. 4A). Furthermore, in SKOV-3 cells, the apoptosis rate was increased from 8.76% to 19.09% by transfection of si-HCP5. In HEY cells, the apoptosis rate was increased from 9.43% to 18.42% by transfection of si-HCP5. Moreover, in both SKOV-3 cells and HEY cells, more cells exhibited G0/G1 arrest and fewer cells exhibited S-phase arrest in the si-HCP5-transfected groups than in the si-NC-transfected groups (Fig. 4C). In addition, in both SKOV-3 cells and HEY cells, the levels of cleaved caspase-3 and cleaved caspase-9 were found to be significantly increased in the si-HCP5-transfected groups (Fig. 4D).

### Silencing of HCP5 Inhibited the Migration of OC Cells

**Fig. 5:**
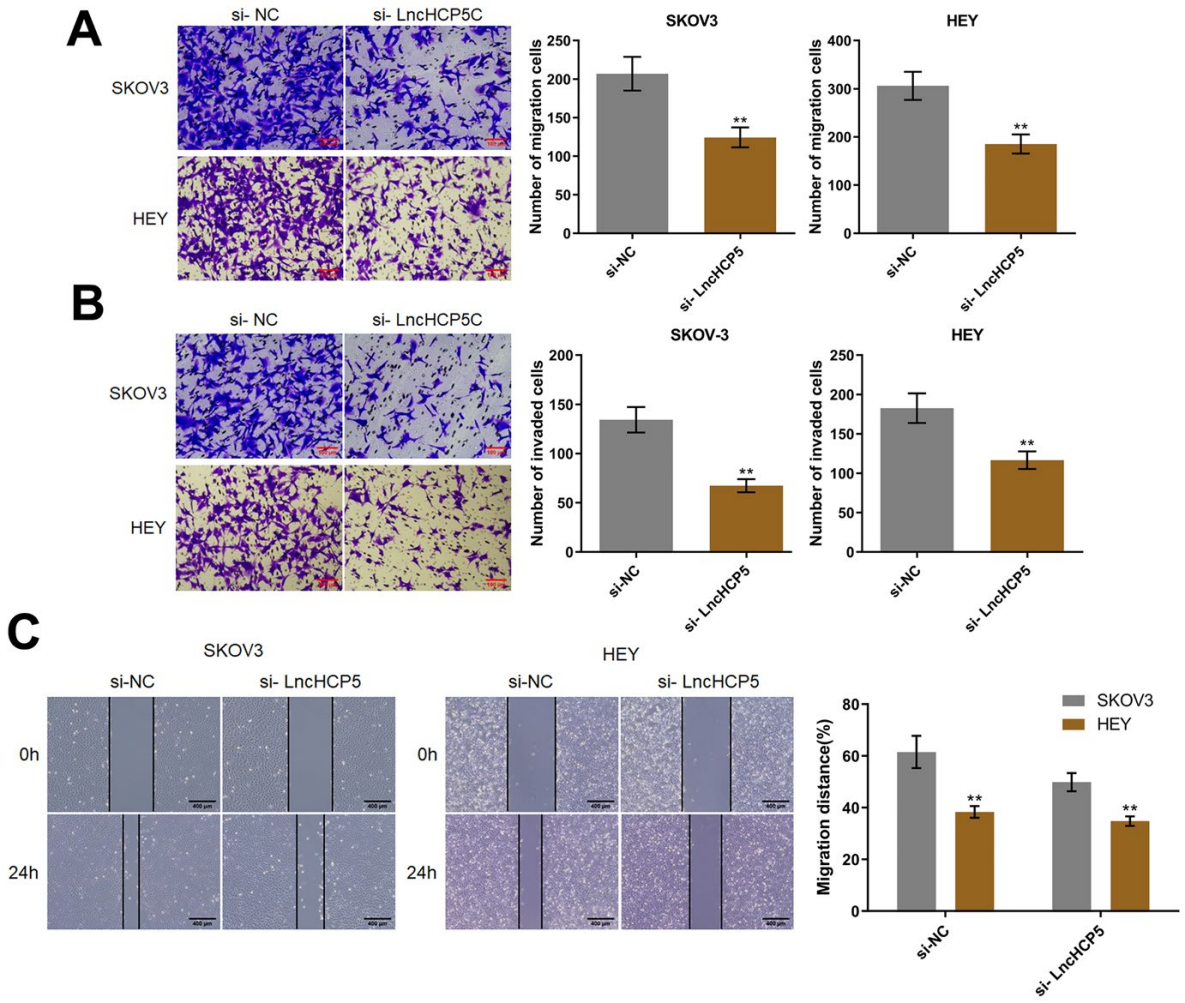
The migration of OC cells was inhibited by silencing of HCP5. The migration (**A**) and invasion (**B**) of SKOV-3 and HEY cells were evaluated by Transwell assays. **C** The migration distance was evaluated by a wound healing assay (**p < 0.01 vs. si-NC)

In SKOV-3 cells, the number of migrated cells was reduced from 207 to 124 by si-HCP5 transfection, while in HEY cells, the numbers of migrated cells in the si-NC and si-HCP5 groups were 306 and 185, respectively (Fig. 5A). Furthermore, the number of invaded SKOV-3 cells was markedly decreased from 134 to 67 by si-HCP5 transfection, while the number of invaded HEY cells was significantly reduced from 183 to 117 by si-HCP5 transfection (Fig. 5B). Moreover, in the wound healing assay, the migration distance of SKOV-3 cells decreased from 61.5% to 38.4% by si-HCP5, while the migration distance of HEY cells in the si-NC and si-HCP5 groups was 49.9% and 34.8%, respectively (Fig. 5C).

### Silencing of HCP5 Suppressed the Migration and Growth of OC Cells In Vivo

**Fig. 6:**
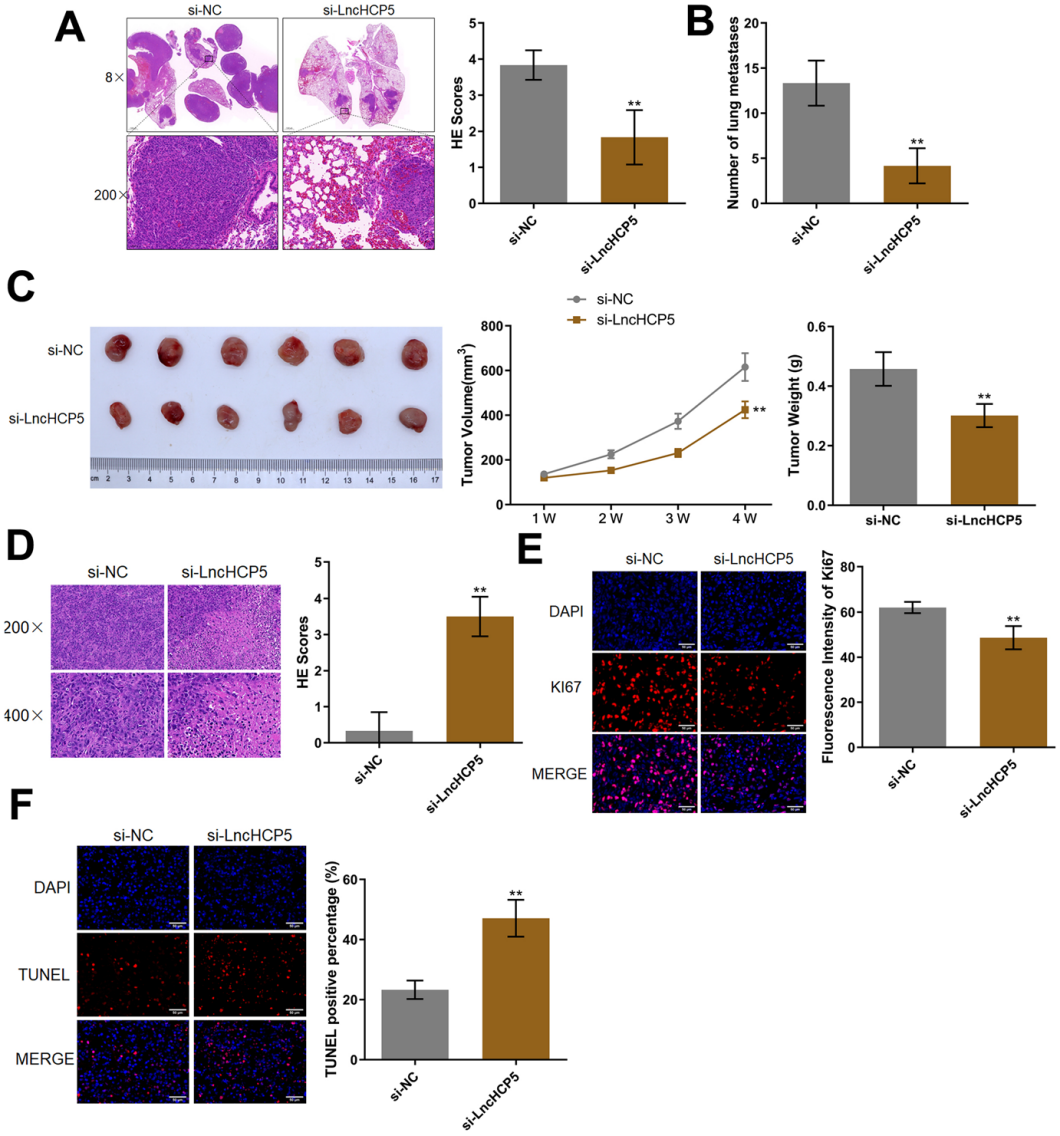
Silencing of HCP5 suppressed the migration and growth of OC cells in vivo. **A** The pathological state of lung tissues was evaluated by HE staining. **B** Lung metastases were counted. **C** Images of tumor tissues, a tumor volume curve, and the weights of tumor tissues are shown. **D** The pathological changes in tumor tissues were detected by HE staining. **E** The expression of Ki67 was visualized using immunofluorescence staining. **F** The apoptotic status in tumor tissues was evaluated by a TUNEL assay (**p < 0.01 vs. si-NC)

To verify that HCP5 influences the growth of OC tumors, si-HCP5-transfected SKOV-3 cells were implanted into nude mice to assess growth and metastasis in vivo. As shown in Fig. 6A, in the si-NC group, atypical cells were observed, with dark nuclear and cytoplasmic staining, an increased nuclear volume, and an increased nuclear/cytoplasmic ratio. In the si-HCP5 group, the number of atypical cells was reduced. Furthermore, the number of metastases in lung tissues was found to be significantly reduced by HCP5 silencing (Fig. 6B). Moreover, compared to those in the si-NC group, the tumor volume and tumor weight in the si-HCP5 group were notably decreased in the SKOV-3 xenograft model (Fig. 6C). Furthermore, in the si-NC group, the glands in the tumor were irregular in shape, with epithelial atypia and pathological mitoses, and these abnormalities were markedly attenuated by HCP5 silencing, along with the increased pathological changes in tumor tissues (Fig. 6D). The fluorescence intensity of Ki67 in tumor tissues was found to be significantly reduced by HCP5 silencing (Fig. 6E). Moreover, the percentage of TUNEL-positive cells in tumor tissues was found to be notably increased by HCP5 silencing (Fig. 6F).

### HCP5 Interacted with PTBP1 in OC Cells

**Fig. 7:**
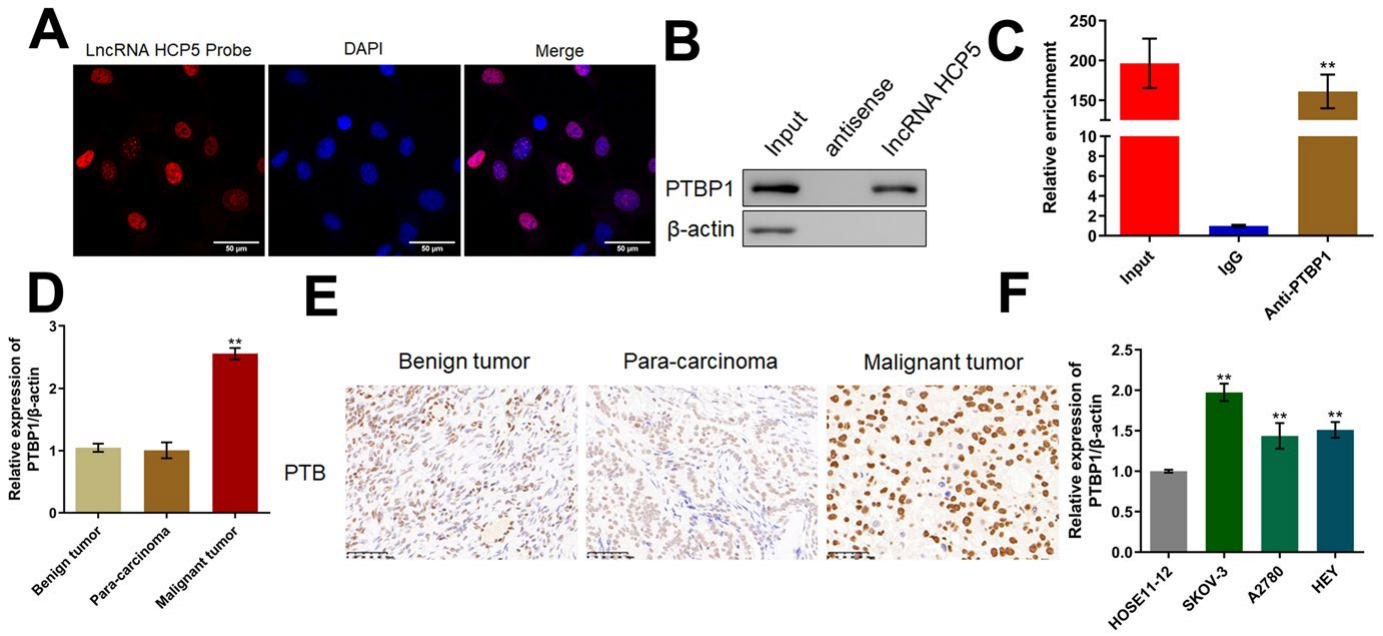
HCP5 interacted with PTBP1 in OC cells. **A** The localization of HCP5 in OC cells was determined by FISH. **B** The binding between HCP5 and PTBP1 was checked by an RNA pull-down assay. **C** The interaction between HCP5 and PTBP1 was evaluated by an RNA immunoprecipitation assay (**p < 0.01 vs. IgG). **D** The expression of PTBP1 in benign tumor tissues, malignant tumor tissues, and paracarcinoma tissues was measured by RT‒PCR (**p < 0.01 vs. benign tumor tissues). **E** The expression of PTBP1 in benign tumor tissues, malignant tumor tissues, and paracarcinoma tissues was evaluated by immunohistochemical staining. The expression of PTBP1 in HOSE11-12, SKOV-3, A2780, and HEY cells was measured by RT‒PCR (**p < 0.01 vs. HOSE11-12)

Several targets of HCP5 have been reported and include PSMB8 (Lei et al. 2020), DDX21 (Wang et al. 2021), and YB1 (Wang et al. 2020a). Furthermore, according to the StarBase database (https://pubmed.ncbi.nlm.nih.gov/24297251/), 4 target sites for binding between HCP5 and PTBP1 were identified. As shown in Fig. S1, compared to that in the si-NC-transfected counterparts, the PTBP1 level was markedly reduced in si-LncHCP5-transfected SKOV-3 cells, with minor changes in the levels of PSMB8, DDX21, and YB1. We suspected that PTBP1 might be the main target of HCP5 in OC cells. To determine whether PTBP1 is a potential target of HCP5 in OC cells, FISH, RNA pull-down, and RNA immunoprecipitation assays were conducted. First, in the FISH assay, HCP5 was found to be localized in the nucleus (Fig. 7A). Furthermore, in the RNA pull-down assay, binding between HCP5 and PTBP1 was detected (Fig. 7B). In the RNA immunoprecipitation assay, HCP5 was found to be enriched by the antibody against PTBP1 (Fig. 7C). To explore the potential function of PTBP1 in OC, the PTBP1 level was measured in benign tumor, malignant tumor and paracarcinoma tissues. As shown in Fig. 7D, compared to that in benign tumor tissues, the PTBP1 level was markedly increased in malignant tumor tissues of OC patients and slightly changed in paracarcinoma tissues of OC patients. The results of the immunohistochemical assay also confirmed that PTBP1 was upregulated in malignant tumor tissues compared to paracarcinoma tissues and benign tumor tissues (Fig. 7E). Furthermore, compared to that in HOSE11-12 cells, the PTBP1 level was markedly increased in SKOV-3, A2780, and HEY cells, with the highest level observed in SKOV-3 cells (Fig. 7F).

### The Influence of HCP5 on OC Cells was Abolished by Knockdown of PTBP1

**Fig. 8:**
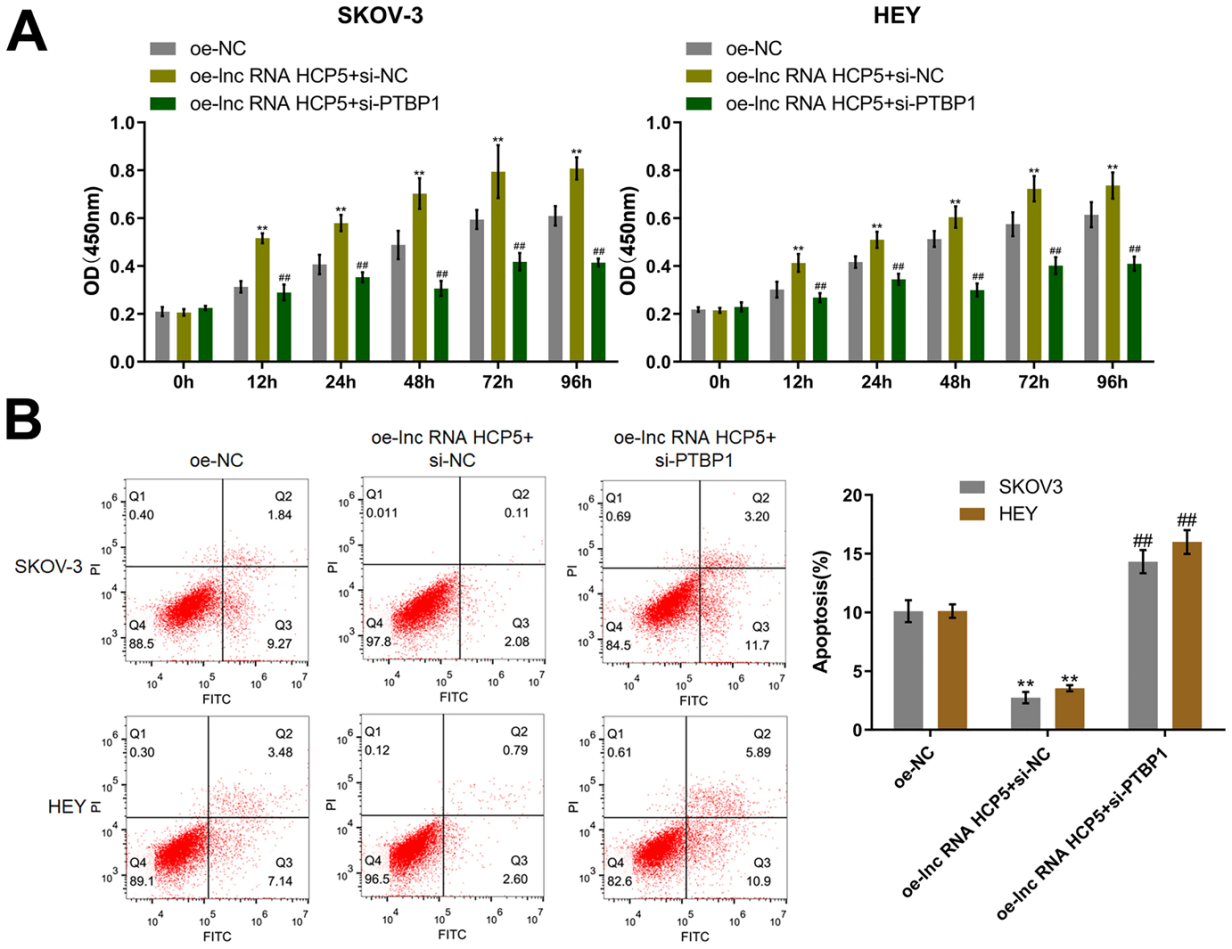
Silencing of PTBP1 abolished the influence of HCP5 on OC cells. **A** The viability of SKOV-3 and HEY cells was checked by a CCK-8 assay. **B** Apoptosis in SKOV-3 and HEY cells was detected by flow cytometry (**p < 0.01 vs. oe-NC, ^##^*p* < 0.01 vs. oe-lnc RNA HCP5+si-NC)

To confirm the function of the HCP5/PTBP1 axis in OC cells, HCP5-overexpressing OC cells were established and were then transfected with si-PTBP1. In both SKOV-3 and HEY cells, compared to transduction of oe-NC, cell viability was markedly increased by transduction of oe-lnc RNA HCP5, and this effect was significantly attenuated by cotransfection of si-PTBP1 (Fig. 8A). Furthermore, the apoptosis rate of SKOV-3 cells was markedly reduced from 11.11% to 2.19% transduction of by oe-lnc RNA HCP5 but was significantly increased to 14.90% by si-PTBP1. Furthermore, the apoptosis rates of HEY cells in the oe-NC, oe-lnc RNA HCP5 + si-NC, and oe-lnc RNA HCP5 + si-PTBP1 groups were 10.62%, 3.39%, and 16.79%, respectively (Fig. 8B).

## Discussion

OC is insidious and often asymptomatic before metastasis. Seventy percent of women are diagnosed with ovarian cancer at an advanced stage, and complete surgical resection of OC tumors is difficult. The sixth most common tumor and one of the three most common malignant tumors in female reproductive organs, OC has the highest mortality among gynecological tumors and is considered the deadliest gynecological malignancy (Asphaug and Melberg 2019; Slomian et al. 2019). Epithelial OC is the most common type of OC, accounting for approximately 85‒90% of OC cases; its 5-year survival rate is approximately 30‒40%, and it has the highest mortality rate among gynecological malignancies. These epithelial tumors originate from the germinal epithelium of the ovarian surface, which is the coelomic epithelium covering the urogenital ridge during the embryonic stage. These epithelia retain undifferentiated immature cells with multilineage differentiation potential, and gene mutations might occur to facilitate the progression of malignant tumors (Zhang et al. 2022; Stewart et al. 2019). Therefore, the search for specific genes related to OC has become the key to improving the clinical diagnosis and treatment of OC. LncRNAs have become a research hotspot in recent years and are involved in the occurrence and development of multiple tumors. LncRNAs lack the ability to encode proteins and are localized in either the nucleus or cytoplasm, which allows them to regulate the expression of genes at various levels (such as the epigenetic, transcriptional, and posttranscriptional levels) (Lin et al. 2020). A large number of studies have reported that the expression levels of specific lncRNAs in tumor cells are abnormally altered, and such changes in expression levels are used as diagnostic markers and potential drug targets for cancers (Bhan et al. 2017; Ashrafizadeh et al. 2022). Herein, HCP5 was found to be markedly upregulated in malignant OC tumor tissues and OC cell lines, a pattern also observed in lung cancer (Li et al. 2020), prostate cancer (Hu and Lu 2020), and breast cancer (Wang et al. 2019). A siRNA-targeting HCP5 was utilized to knock down HCP5 in OC cells, which resulted in suppressed proliferation, inhibited migration, and increased apoptosis in OC cells. Chen (Chen et al. 2020) and Yun (Yun et al. 2019) also reported the oncogenic function of HCP5 in granulosa-like tumor cells and colon tumor cells, respectively. Furthermore, the inhibitory function of si-HCP5 against OC metastasis and growth was verified in a xenograft model established with OC cells, the results of which were in line with the research of HCP5 in nasopharyngeal carcinoma (Miao et al. 2022) and in triple-negative breast cancer (Wang et al. 2019). Although a previous study reported the ceRNA function of HCP5 in OC (Wang et al. 2020b), HCP5 was observed to be mainly localized in the nucleus, not in the cytoplasm. We suspected that HCP5 might function in OC by directly interacting with its target protein.

Polypyrimidine tract-binding (PTB) protein is an RNA-binding alternative splicing regulatory protein (Arake de Tacca et al. 2019) and a member of the heterogeneous nuclear ribonucleoprotein (hnRNP) family of alternative splicing regulatory proteins. As a splicing regulator, PTBP1 is involved in the alternative splicing (AS) of multiple genes (Zhu et al. 2020). PTBP1, also known as hnRNPL, is mainly involved in the regulation of gene transcription, mediating the regulation of alternative splicing events in tumor invasion-related genes. PTBP1 is involved in the formation, invasion, and metastasis of bladder cancer, pancreatic cancer, lung cancer, ovarian cancer, and other cancers (Zhu et al. 2020). Athena claimed that PTBP1 exerted a protumorigenic effect by regulating the secretion of inflammatory mediators (Georgilis et al. 2018). Ji claimed that PTBP1 was an important factor involved in the oncogenic function of the lncRNA LINREP in glioblastoma progression (Ji et al. 2023). Herein, PTBP1 was found to be markedly upregulated in malignant OC tumor tissues, implying a potential oncogenic function of PTBP1 in OC. According to the prediction results of the StarBase database and the results of RNA pull-down and RNA immunoprecipitation assays, an interaction occurs between HCP5 and PTBP1. Furthermore, in HCP5-overexpressing OC cells, the impact of HCP5 on cell proliferation and apoptosis was significantly attenuated by the knockdown of PTBP1, suggesting that PTBP1 is downstream of HCP5 and mediates the oncogenic function of HCP5 in OC.

Although the present study preliminarily revealed the function of HCP5 in OC, there are several limitations. First, PTBP1 was chosen as the potential target of HCP5 based only on the StarBase prediction. Other potential targets predicted in StarBase, such as eIF4AIII, UPF1, and U2AF65, should be investigated to determine whether they are indeed targets of HCP5. Furthermore, the specific binding site of HCP5 to the PTBP1 protein should be explored using bioinformatic methods. Last, the impact of PTBP1 knockdown on the oncogenic function of HCP5 will be further verified in a xenograft model in future work.

Collectively, these results indicate that HCP5 facilitates the progression of OC by interacting with the PTBP1 protein.

### Supplementary Information

Below is the link to the electronic supplementary material.

Supplementary file1 (JPG 411 kb)

The PTBP1 level was markedly decreased in si-LncHCP5-transfected SKOV3 cells. The expression of PTBP1, PSMB8, DDX21, and YB1 in SKOV3 cells was measured using Western blotting (**p<0.01 vs. si-NC).

## Data Availability

All data are available from the corresponding author if requested by the journal or the readers.
