# Supplementary figures and images for: LncRNA HCP5 Facilitates the Progression of Ovarian Cancer by Interacting with the PTBP1 Protein
Source: Biochem Genet. 2023 Dec 10;62(4):3136–54. doi: 10.1007/s10528-023-10558-8 (PMC11289333; doi:10.1007/s10528-023-10558-8)

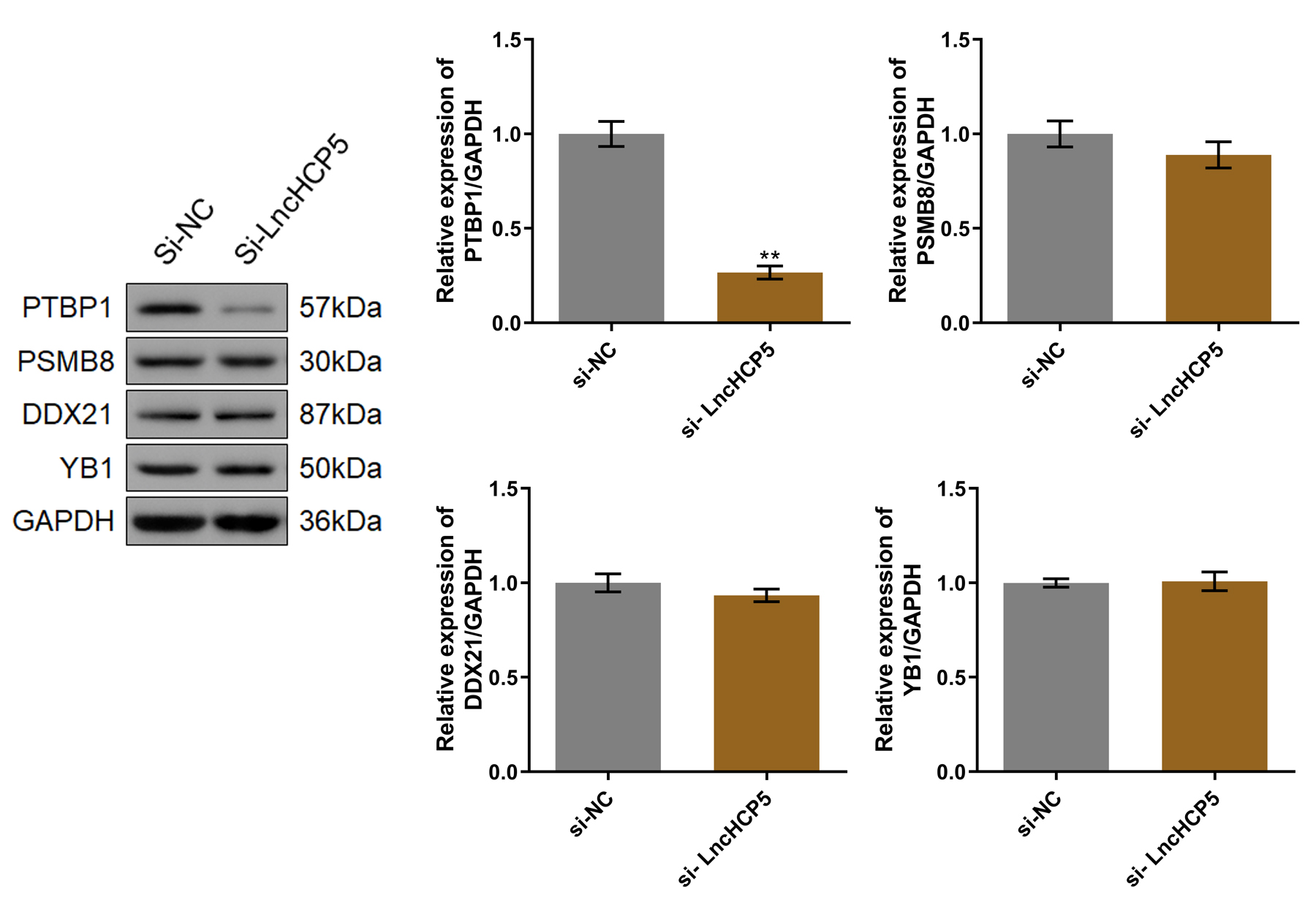

Supplement: Supplementary file 1 — Supplementary file1 (JPG 411 kb) [file 10528_2023_10558_MOESM1_ESM.jpg]
